# Diabetic retinopathy as a potential marker of Parkinson’s disease: a register-based cohort study

**DOI:** 10.1093/braincomms/fcab262

**Published:** 2021-11-08

**Authors:** Maria E C Larsen, Anne S Thykjaer, Frederik N Pedersen, Sören Möller, Caroline S Laugesen, Nis Andersen, Jens Andresen, Javad Hajari, Steffen Heegaard, Kurt Højlund, Ryo Kawasaki, Katja C Schielke, Katrine H Rubin, Morten Blaabjerg, Lonny Stokholm, Jakob Grauslund

**Affiliations:** 1 Department of Ophthalmology, Odense University Hospital, 5000 Odense, Denmark; 2 Department of Clinical Research, University of Southern Denmark, 5000 Odense, Denmark; 3 Steno Diabetes Center Odense, Odense University Hospital, 5000 Odense, Denmark; 4 OPEN—Open Patient data Explorative Network, Odense University Hospital and University of Southern Denmark, 5000 Odense, Denmark; 5 Department of Ophthalmology, Zealand University Hospital Roskilde, 4000 Roskilde, Denmark; 6 Organization of Danish Practicing Ophthalmologists, 2100 Copenhagen, Denmark; 7 Department of Ophthalmology, Rigshospitalet-Glostrup, 2600 Glostrup, Denmark; 8 Department of Clinical Medicine, University of Copenhagen, 2200 Copenhagen, Denmark; 9 Department of Vision Informatics, University of Osaka, Osaka 656-0871, Japan; 10 Department of Ophthalmology, Aalborg University Hospital, 9000 Aalborg, Denmark; 11 Department of Neurology, Odense University Hospital, 5000 Odense, Denmark

**Keywords:** diabetic retinopathy, diabetes, epidemiology, neurodegeneration, Parkinson’s disease

## Abstract

Neurodegeneration is an early event in the pathogenesis of diabetic retinopathy, and an association between diabetic retinopathy and Parkinson’s disease has been proposed. In this nationwide register-based cohort study, we investigated the prevalence and incidence of Parkinson’s disease among patients screened for diabetic retinopathy in a Danish population-based cohort. Cases (*n* = 173 568) above 50 years of age with diabetes included in the Danish Registry of Diabetic Retinopathy between 2013 and 2018 were matched 1:5 by gender and birth year with a control population without diabetes (*n* = 843 781). At index date, the prevalence of Parkinson’s disease was compared between cases and controls. To assess the longitudinal relationship between diabetic retinopathy and Parkinson’s disease, a multivariable Cox proportional hazard model was estimated. The prevalence of Parkinson’s disease was 0.28% and 0.44% among cases and controls, respectively. While diabetic retinopathy was not associated with present (adjusted odds ratio 0.93, 95% confidence interval 0.72–1.21) or incident Parkinson’s disease (adjusted hazard ratio 0.77, 95% confidence interval 0.56–1.05), cases with diabetes were in general less likely to have or to develop Parkinson’s disease compared to controls without diabetes (adjusted odds ratio 0.79, 95% confidence interval 0.71–0.87 and adjusted hazard ratio 0.88, 95% confidence interval 0.78–1.00). In a national cohort of more than 1 million persons, patients with diabetes were 21% and 12% were less likely to have prevalent and develop incident Parkinson’s disease, respectively, compared to an age- and gender-matched control population without diabetes. We found no indication for diabetic retinopathy as an independent risk factor for incident Parkinson’s disease.

## Teaser

In this register-based cohort study investigating the association between diabetic retinopathy and Parkinson’s disease, Larsen et al. report that diabetic retinopathy does not increase the risk of having or developing Parkinson’s disease; on the contrary, diabetes *per se* seems to have a protective effect.

## Graphical Abstract

**Graphical Abstract:**
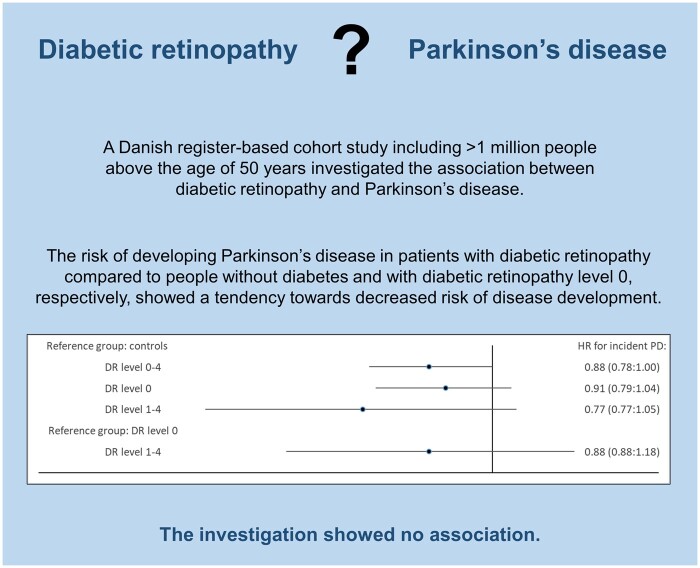

## Introduction

Diabetic retinopathy is the most common complication in diabetes^1–4^ and a leading cause of visual impairment.^5^^,^^6^ It has previously been considered a microvascular disease, but recent evidence identifies retinal neurodegeneration as an early pathogenic event.^7–9^

Parkinson’s disease is the second most common neurodegenerative disorder, affecting one in 1000 people aged >70 years,^10^ and it is estimated that approximately 8000 people live with Parkinson’s disease in Denmark.^11^ It is a complex and multifactorial disease characterized by motor symptoms such as bradykinesia, rigidity and resting tremor caused by a loss of dopaminergic neurons in the substantia nigra.^12^^,^^13^ The disease is progressive, and currently no curative treatment is available.^14^ However, early diagnosis and timely symptomatic treatment have shown to have a beneficial effect on the progression of the disease.^15^

In the retina, dopamine serves e.g. as a chemical messenger for light adaptation and plays a role in the circadian rhythmicity.^16^ An association between diabetic retinopathy and Parkinson’s disease has been proposed, based on the assumption that both can be related to an abnormal dopaminergic system.^8^ Studies have shown that diabetes reduces dopamine levels in the retina of rodents,^17^ and that medical treatment with levodopa can reverse retinal dysfunction in patients without diabetic retinopathy.^18^ A Korean study found that patients with type 2 diabetes and diabetic retinopathy had an increased risk of developing Parkinson’s disease compared to patients with diabetes and no diabetic retinopathy.^19^ We aim to evaluate and further explore this finding in a Danish register-based cohort, by following patients with any type of diabetes and adding relevant information to the analyses, such as duration of diabetes, in order to understand deeply the potential role of diabetic retinopathy.

## Materials and methods

The study was conducted as a matched cohort study based on data from several Danish registries. The first part of the study investigated the prevalence of Parkinson’s disease among patients with diabetes and diabetic retinopathy at baseline, while the second part investigated the incidence of Parkinson’s disease in a 5-year prospective cohort.

### Data sources

In Denmark, healthcare is government-funded and freely accessible for all residents, and information regarding diagnoses and treatments is registered and kept in comprehensive national registries.^20^ In this study, data from four different registries were used: the Danish Registry of Diabetic Retinopathy, the Danish National Patient Register, the Danish National Prescription Registry and the Danish Civil Registration System (for extended description, see Appendix I in the Supplementary material).

The Danish Registry of Diabetic Retinopathy is a national clinical quality database that collects data on all patients above 18 years of age diagnosed with diabetes, who attend the national screening programme for diabetic eye disease.^21^ The registry includes data on all patients screened at selected departments of ophthalmology and by practicing ophthalmologists. The screening programme is free of charge, and all patients with diabetes are encouraged to participate. The grading of diabetic retinopathy is done according to the International Clinical Diabetic Retinopathy Disease Severity Scale, which contains five stages: level 0 (no diabetic retinopathy), 1–3 (mild, moderate and severe non-proliferative diabetic retinopathy) or 4 (proliferative diabetic retinopathy).^22^

The Danish National Patient Register contains information on all hospital contacts,^23^ and individuals are diagnosed and coded according to the 10th version of the International Classification of Diseases system.^24^

The National Prescription Registry contains information on prescribed medication redeemed at community pharmacies in Denmark.^20^ The prescribed medication is classified in accordance with the Anatomical Therapeutic Chemical Classification System.^25^

From the Danish Civil Registration System we retrieved information on gender, birth year, marital and vital status.^26^

### Study population

**Figure 1:**
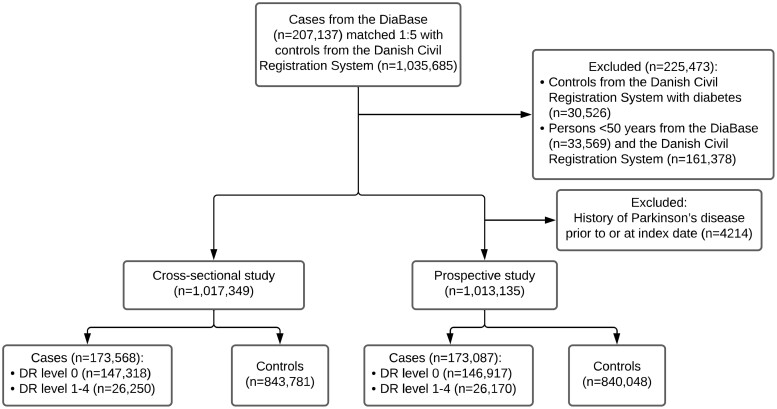
**Flowchart of the selection of study participants for the different parts of the study.** DR, diabetic retinopathy.

Cases were defined as patients with diabetes ≥50 years of age screened for diabetic retinopathy between 2013 and 2018. They were extracted from The Danish Registry of Diabetic Retinopathy and randomly matched 1:5 according to gender and birth year with background population extracted from the Danish Civil Registration System. Controls subsequently identified with diabetes were excluded. Index date was defined as the date of the first reported screening episode registered in The Danish Registry of Diabetic Retinopathy, and controls were assigned identical index dates to their corresponding cases (Fig.  1). The analyses were conducted on a population ≥50 years of age at index date to avoid immortal time bias, as Parkinson’s disease in its common sporadic form is rare before the age of 50 years, and as young people often get familial forms.^15^^,^^27^

A combination of ICD-10 codes for diabetes and ATC codes for insulin and glucose lowering drugs were used to determine the type of diabetes among cases, and to exclude patients with diabetes among controls (Supplementary Table 1). The duration of diabetes was defined as the difference between the first ICD-10 code or prescription for diabetes and index date, whichever occurred first.

Patients were defined as having Parkinson’s disease when given a primary and/or secondary diagnostic code of Parkinson’s disease (ICD-10 = G209). To increase the validity of the diagnosis, ≥2 redeemed prescriptions of antiparkinsonian medication [including levodopa (ATC=N04BA), dopamine agonists (ATC=N04BC) and MAO-B inhibitors (ATC=N04BD)] the year following the diagnosis were required. We excluded patients with (i) any previous record of atypical or secondary forms of parkinsonism (ICD-10 = G21), (ii) a diagnosis of essential tremor (ICD-10 = G20) or (iii) the use of antipsychotic medicine (ATC=N05A*) at the time of receiving the Parkinson’s disease diagnosis (redeemed prescription <180 days prior to the date of diagnosis), because of the risk of extrapyramidal side effects. For the prospective part of the study, all patients defined as having Parkinson’s disease before/at index date were excluded to ensure only incident cases of Parkinson’s disease were counted as outcome. Charlson Comorbidity Index was used to describe and control for comorbidity among cases and controls. The possible confounding effect of medication including antihypertensive drugs and cholesterol lowering drugs was adjusted for in the multivariable analyses.

### Statistical analyses

The primary endpoints were (i) multivariable odds ratio for prevalent Parkinson’s disease at baseline and (ii) multivariable HR for 5-year incident Parkinson’s disease, all among patients with diabetes and no or any level of diabetic retinopathy, compared to a diabetes-free background population. As a secondary outcome, patients with diabetic retinopathy level 1–4 were compared to patients with diabetic retinopathy level 0. Diabetic retinopathy level 1–4 was pooled in order to obtain statistical power in the analyses. Cases were followed until the first registration of Parkinson’s disease, until they died, emigrated or reached the date of end of follow-up (31 December 2018), whichever occurred first. For the test of significance, we used Pearson’s Chi-squared test while for the continuous variables, the *k*-sample equality-of-median test was used. *P*-value <0.05 or confidence interval (CI) not including 1 were considered statistically significant. As a supplementary analysis, the inverse relationship between diabetic retinopathy and Parkinson’s disease was investigated in a prospective analysis.

As information on current smoking status was unavailable and as smoking is known to be a protective factor for Parkinson’s disease,^28^ we created a variable for smoking based on characteristics of smoking, by compiling ICD-10 and ATC codes related to smoking (Supplementary Table 2), as done in another Danish register-based study.^29^ The variable was used to conduct a sensitivity analysis to evaluate the effect of the potential difference in smoking habits among persons with and without diabetes, on the development of Parkinson’s disease.^30^^,^^31^

Data management and analyses were done using Stata version 16.1.

### Data availability

Results were based on data from Danish national registries and individual data cannot be shared. Authorization to data access is given by The Danish National Health Data Authority and requires permission from the Danish Data Protection Agency.

## Results

The analyses were based on a study population of 1 017 349 individuals >50 years of age, of which 173 568 had diabetes and 843 781 did not. Table  1 describes the clinical characteristics of the case population at index date at an overall and according to level of diabetic retinopathy. The mean age was 68.1 years, and 56.7% were males. In general, a more severe degree of diabetic retinopathy was associated with a higher Charlson Comorbidity Index score (*P *<* *0.001) as well as a higher percentage of cases using anti-diabetic, antihypertensive and cholesterol lowering drugs (*P *<* *0.001). The probability of not being married increased with the severity of diabetic retinopathy.

**Table 1:** Characteristics of patients ≥50 years of age with diabetes at the first occurrence in the DiaBase according to level of diabetic retinopathy

		Level of DR					
	Overall	Level 0	Level 1	Level 2	Level 3	Level 4	*P*-value
Number of patients, *n*	173 568	147 318	16 355	5136	759	4 000	
Gender, *n* (%) male	98 438 (56.7)	82 515 (56.0)	9 775 (59.8)	3 174 (61.8)	512 (67.5)	2 462 (61.5)	<0.001
Age, years (IQR)	68.1 (60.8–74.5)	68.3 (61.1–74.6)	67.6 (60.1–74.5)	66.1 (58.8–72.8)	62.6 (56.5–69.9)	66.0 (58.4–72.9)	<0.001
Type of diabetes, *n* (%)							<0.001
Type 1 diabetes	6616 (3.8)	3217 (2.2)	1 941 (11.9)	497 (9.7)	53 (7.0)	908 (22.7)	
Type 2 diabetes	137 337 (79.1)	125 216 (85.0)	8 553 (52.3)	2 369 (46.1)	336 (44.3)	863 (21.6)	
Unknown	29 615 (17.1)	18 885 (12.8)	5 861 (35.8)	2 270 (44.2)	370 (48.7)	2 229 (55.7)	
Duration of diabetes, years (IQR)^a^							<0.001
Type 1 diabetes	19.8 (13.8–21.5)	16.8 (6.8–20.9)	20.2 (19.3–21.8)	20.4 (19.2–22.2)	20.2 (19.0–21.1)	20.6 (19.6–22.3)	
Type 2 diabetes	5.7 (2.3–10.2)	5.3 (2.2–9.4)	11.0 (5.8–15.7)	11.7 (6.1–16.4)	12.6 (6.3–17.0)	14.7 (9.2–19.2)	
Unknown	14.5 (9.3–19.3)	12.0 (7.4–16.9)	18.2 (13.9–20.1)	18.1 (13.6–20.2)	17.6 (13.8–20.1)	19.9 (18.1–21.2)	
Marital status, *n* (%)							<0.001
Never married	17 704 (10.2)	14 517 (9.9)	1 827 (11.2)	659 (12.8)	107 (14.1)	594 (14.8)	
Married or living with someone	103 362 (59.6)	88 260 (59.9)	9 515 (58.2)	2 920 (56.9)	412 (54.3)	2 255 (56.4)	
Widowed or divorced	52 502 (30.2)	44 541 (30.2)	5 013 (30.7)	1 557 (30.3)	240 (31.6)	1 151 (28.8)	
Charlson Comorbidity Index score, *n* (%)							<0.001
0 (low)	122 478 (70.6)	108 648 (73.8)	9 414 (57.6)	2 654 (51.7)	350 (46.1)	1 412 (35.3)	
1 (moderate low)	23 244 (13.4)	16 199 (11.0)	3 775 (23.1)	1 443 (28.1)	241 (31.8)	1 586 (39.6)	
2 (moderate high)	17 386 (10.0)	14 537 (9.9)	1 695 (10.4)	545 (10.6)	93 (12.3)	516 (12.9)	
≥3 (high)	10 460 (6.0)	7 934 (5.4)	1 471 (9.0)	494 (9.6)	75 (9.9)	486 (12.2)	
Use of medication, *n* (%)							
Insulin	49 925 (28.8)	31 876 (21.6)	10 295 (62.9)	3 736 (72.7)	567 (74.7)	3 451 (86.3)	<0.001
Glucose lowering treatment, excluding insulins	137 758 (79.4)	120 996 (82.1)	11 109 (67.9)	3 494 (68.0)	519 (68.4)	1 640 (41.0)	<0.001
Antihypertensive drugs	141 563 (81.6)	118 815 (80.7)	14 052 (85.9)	4 411 (85.9)	656 (86.4)	3 629 (90.7)	<0.001
Cholesterol lowering drugs	137 124 (79.0)	116 082 (78.8)	13 167 (80.5)	4 013 (78.1)	588 (77.5)	3 274 (81.8)	<0.001
Parkinson's disease, *n* (%)	481 (0.3)	401 (0.3)	46 (0.3)	>10 (0.3)	<5	17 (0.4)	0.132

Results are given as number (%) or median (IQR).

a Duration of diabetes was calculated only for patients with at least one International Classification of Diseases version 10 code for diabetes or one Anatomical Therapeutic Chemical Classification codes for treatment of diabetes.

DR, diabetic retinopathy; IQR, interquartile range; *n*, count; N/A, not applicable.

At index date, 481 cases and 3733 controls had a diagnosis of Parkinson’s disease (Table  2). Individuals with Parkinson’s disease were older and predominantly males compared to individuals without Parkinson’s disease. The same group of people were also more likely to be married or living with someone (*P *=* *0.002 and <0.001). Cases with Parkinson’s disease had a smaller consumption of glucose lowering drugs excluding insulins, antihypertensive drugs and cholesterol lowering drugs compared to cases without Parkinson’s disease, as well as a longer duration of diabetes (*P *<* *0.001).

**Table 2:** Differences between patients ≥50 years with and without Parkinson’s disease at index date

		Cases			Controls	
		Persons with PD	Persons without PD	*P*-value	Persons with PD	Persons without PD	*P*-value
Number of patients, *n*		481	173 087		3 733	840 048	
Gender, *n* (%) male		337 (70.1)	98 101 (56.7)	<0.001	2 522 (67.6)	474 731 (56.5)	<0.001
Age, years (IQR)		74.1 (68.8–79.3)	68.1 (60.8–74.5)	<0.001	75.4 (70.180.2)	68.0 (60.7–74.4)	<0.001
Type of diabetes, *n* (%)				<0.001			N/A
Type 1 diabetes		11 (2.3)	6 605 (3.8)		N/A	N/A	
Type 2 diabetes		349 (72.6)	136 988 (79.1)		N/A	N/A	
Unknown		121 (25.2)	29 494 (17.0)		N/A	N/A	
Marital status, *n*(%)				0.002			<0.001
Never married		26 (5.4)	17 678 (10.2)		199 (5.3)	79 568 (9.5)	
Married or living with someone		308 (64.0)	103 054 (59.5)		2 425 (65.0)	526 032 (62.6)	
Widowed or divorced		147 (30.6)	52 355 (30.2)		1 109 (29.7)	234 448 (27.9)	
Charlson Comorbidity Index score, *n* (%)				<0.001			<0.001
0 (low)		275 (57.2)	122 203 (70.6)		2 771 (74.2)	707 384 (84.2)	
1 (moderate low)		92 (19.1)	23 152 (13.4)		186 (5.0)	43 281 (5.2)	
2 (moderate high)		79 (16.4)	17 307 (10.0)		609 (16.3)	67 395 (8.0)	
≥3 (high)		35 (7.3)	10 425 (6.0)		167 (4.5)	21 988 (2.6)	
Use of medication, *n* (%)							
Insulin		161 (33.5)	49 764 (28.8)	0.022	N/A	N/A	N/A
Glucose lowering treatment, excluding insulins		348 (72.3)	137 410 (79.4)	<0.001	N/A	N/A	N/A
Antihypertensive drugs		369 (76.7)	141 194 (81.6)	0.006	1 603 (42.9)	362 277 (43.1)	0.820
Cholesterol lowering drugs		357 (74.2)	136 767 (79.0)	0.010	983 (26.3)	227 734 (27.1)	0.287
Level of DR, *n* (%)				0.392			N/A
0		401 (83.4)	146 917 (84.9)		N/A	N/A	
1		46 (9.6)	16 309 (9.4)		N/A	N/A	
2		>10 (3.3)	5 120 (3.0)		N/A	N/A	
3		<5	758 (0.4)		N/A	N/A	
4		17	3 983 (2.3)		N/A	N/A	
Level of DR given by the worse eye							

Results are given as number (%) or median (IQR).

DR, diabetic retinopathy; IQR, interquartile range; *n*, count; N/A, not applicable; PD, Parkinson’s disease.

In the cross-sectional analysis, cases had a lower risk of having Parkinson’s disease (odds ratio 0.79, 95% CI 0.71–0.87) at index date (Table  3) compared to controls. This was true for cases without diabetic retinopathy (odds ratio 0.76, 95% CI 0.68–0.85), but not for cases with diabetic retinopathy level 1–4 (odds ratio 0.93, 95% CI 0.72–1.21).

**Table 3:** Odds ratio with 95% confidence intervals for Parkinson’s disease at index date among patients screened for diabetic retinopathy, compared to a 1:5 age- and gender-matched control population without diabetes

	Cases		Controls		Crude model	Model adjusted for age and gender	Multivariable model^a^
Level of DR	Persons with PD	Persons without PD	Persons with PD	Persons without PD	OR (95%CI)	OR (95%CI)	OR (95%CI)
Overall	481	173 087	3 733	840 048	0.63 (0.57–0.69)	0.62 (0.57–0.68)	0.79 (0.71– 0.87)
Level 0	401	146 917	3 228	713 204	0.60 (0.54–0.67)	0.60 (0.54–0.67)	0.76 (0.68–0.85)
Level 1–4	80	26 170	505	126 844	0.77 (0.61–0.97)	0.76 (0.60–0.97)	0.93 (0.72–1.21)

Results are given as number or OR (95% CI).

a Multivariable logistic regression model adjusted for sex, age, marital status, use of antihypertensive drugs, cholesterol lowering drugs and Charlson Comorbidity Index: myocardial infarction, congestive heart failure, peripheral vascular disease, cerebrovascular disease, chronic pulmonary disease, connective tissue disease and rheumatologic disease, ulcer disease, mild or moderate/severe liver disease, hemiplegia or paraplegia, moderate or severe renal disease, any malignancies (including leukaemia and lymphoma) and acquired immunodeficiency syndrome.

CI, confidence interval; DR, diabetic retinopathy; OR, odds ratio; PD, Parkinson’s disease.

Of the 2305 events of Parkinson’s disease during the 5-year follow-up, 365 occurred in the case group in comparison to 1940 in the control group, showing a trend towards a decreased risk of developing Parkinson’s disease in persons with diabetes (adjusted HR 0.88, 95% CI 0.78–1.00) compared to persons without diabetes (Table  4). Analyses on individuals with diabetic retinopathy level 0 or 1–4 showed no association between diabetic retinopathy and Parkinson’s disease (adjusted HR 0.91, 95% CI 0.79–1.03 and 0.77, 95% CI 0.56–1.05, respectively). Results were not altered, when the effect of smoking was added in a sensitivity analysis (Table  4). In the analysis for the secondary outcome, the effect of diabetic retinopathy was isolated by using cases without diabetic retinopathy as reference. For cases without diabetic retinopathy, 310 developed Parkinson’s disease within 45 229 person-years in comparison to 55 events in 94 798 person-years for cases with diabetic retinopathy (multivariable adjusted HR 0.74, 95% CI 0.54–1.02). In a separate multivariable analysis looking at type 1 and type 2 diabetes individually, no association was found between diabetic retinopathy and Parkinson’s disease, neither in the cross-sectional or prospective part of the study. More specifically, the prospective multivariable analysis for type 1 and type 2 diabetes with diabetic retinopathy level 1–4 showed a hazard ratio of 0.67, 95% CI 0.27–1.64 and 0.84, 95% CI 0.56–1.25, respectively.

**Table 4:** Hazard ratio with 95% confidence intervals for Parkinson’s disease after index date for patients screened for diabetic retinopathy, compared to a 1:5 age- and gender-matched control population without diabetes

	Cases		Controls		Crude model	Model adjusted for age and gender	Multivariable model*	Multivariable model^a^ including smoking HR (95%CI)
Level of DR	Events of PD	Years of risk	Events of PD	Years of risk	HR (95%CI)	HR (95%CI)	HR (95%CI)	
All	365	547 093	1 940	2 658 139	0.91 (0.82–1.02)	0.90 (0.81–1.01)	0.88 (0.78–1.00)	0.88 (0.78–1.00)
0	310	452 295	1 611	2 185 250	0.93 (0.82–1.05)	0.92 (0.81–1.04)	0.91 (0.79–1.04)	0.91 (0.80–1.04)
1–4	55	94 798	329	472 889	0.83 (0.63–1.11)	0.83 (0.63–1.11)	0.77 (0.56–1.05)	0.77 (0.56–1.05)

Results are given as number or HR (95% CI).

a Multivariable logistic regression model adjusted for sex, age, marital status, use of antihypertensive drugs, cholesterol lowering drugs and Charlson Comorbidity Index: myocardial infarction, congestive heart failure, peripheral vascular disease, cerebrovascular disease, chronic pulmonary disease, connective tissue disease and rheumatologic disease, ulcer disease, mild or moderate/severe liver disease, hemiplegia or paraplegia, moderate or severe renal disease, any malignancies (including leukaemia and lymphoma) and acquired immunodeficiency syndrome.

CI, confidence interval; DR, diabetic retinopathy; HR, hazard ratio; PD, Parkinson’s disease.

The risk of 5-year incident diabetic retinopathy did not differ between cases with and without Parkinson’s disease at index date (26 events in 811 person-years versus 10 399 events in 359 302 person-years, multivariable adjusted HR 0.99, 95% CI 0.67–1.45).

## Discussion

Based on data of >1 million Danish citizens above the age of 50 years, we did not find clinical evidence that diabetic retinopathy acts as an independent marker of Parkinson’s disease. In addition, our results indicate that persons with diabetes, attending the eye-screening programme, are less likely to have or to develop Parkinson’s disease.

It was the discovery of the shared pathophysiological characteristics of diabetic retinopathy and Parkinson’s disease that led to the hypothesis of diabetic retinopathy and Parkinson’s disease being positively associated.^7^^,^^8^ To our knowledge, only one other study has been conducted on this association. Lee et al.^19^ concluded in 2017 that patients with diabetic retinopathy had an increased risk of developing Parkinson’s disease (HR 1.75, 95% CI 1.64–1.86). Their case population included >1 million patients with type 2 diabetes and the definition of diabetic retinopathy was based on ICD-10 codes. In our study, we tried to broaden the study population by also including patients with type 1 diabetes as well as separating our study population with diabetes into two groups (no diabetic retinopathy versus diabetic retinopathy level 1–4), as rated by ophthalmologists. Furthermore, we had information on the duration of diabetes and included many comorbidities in our multivariable analysis. Our definition of Parkinson’s disease was narrow to ensure only true persons with Parkinson’s disease were included in the analyses. This might account for fewer events in our cohort in comparison to Lee et al., who were broader in their definition, defining Parkinson’s disease as one ICD-10 code of Parkinson’s disease during hospitalization or two ICD-10 codes in the outpatient clinic. The conflicting results may also be related to national differences such as genetics, demographics and exposures, which are known to constitute risk factors on the development of Parkinson’s disease.^32^

The association between diabetes and Parkinson’s disease is still controversial.^13^^,^^33^ Some studies agree that diabetes is a risk factor for Parkinson’s disease while other studies find no evidence of this association.^34–38^ A meta-analysis from 2021 by Chohan et al.^38^ observed evidence for type 2 diabetes being positively associated with an increased risk of Parkinson’s disease. Nevertheless, we did not find this association in our analysis when investigating the effect of diabetes of all kinds or type 2 diabetes specifically. In a Spanish population-based cross-sectional study investigating the association between diabetes and Parkinson’s disease, no positive association was found.^39^ However, the results did suggest a disease-duration-dependent effect, meaning that patients with long-duration diabetes might have an increased risk of developing Parkinson’s disease. Our results support that persons with Parkinson’s disease generally had a longer duration of diabetes.

As the retina is generally considered as a window to the brain, the idea of exploring the relationship between diabetic retinopathy and neurodegenerative diseases is not unfamiliar. Strong evidence already associates diabetic retinopathy to cognitive impairment and diseases such as Alzheimer disease.^40^^,^^41^ The important task is to recognize the true associations. Our results are valuable as they are based on data of a large national sample population, including almost half of the Danish population above 50 years.^42^ It is not possible to know the exact number of people living with Parkinson’s disease in Denmark. The definition of patients with Parkinson’s disease in this study is inspired by methods used in a report from 2015 by The Danish Parkinson’s Association.^11^ According to a Danish validation study of The Danish National Patient Register , only 82% of patients diagnosed with Parkinson’s disease actually have Parkinson’s disease.^43^ To avoid false positive diagnoses of Parkinson’s disease in our study population, we confirmed the diagnosis with two redeemed prescriptions of antiparkinsonian medication the year following the diagnosis. Few differences in baseline characteristics are found when comparing the Parkinson group and the non-Parkinson group. Patients with Parkinson’s disease generally have a higher Charlson Comorbidity Index score compared to controls, while patients with Parkinson’s disease tend to use less medication. We cannot deduce whether this difference is causally conditioned or if it is due to the presumably stronger hospital connection for patients with Parkinson’s disease, which might give them a greater chance of adequate medical treatment of comorbidities.

The strengths of this study include the large population size and the avoidance of selection bias, as participants from registries do not have to volunteer. Another strength was the access to The Danish Registry of Diabetic Retinopathy, which allowed us to include a case population who with certainty suffered from diabetes. The government-funded eye-screening programme ensures equal possibility of attendance for all levels of society and thereby includes as many events of diabetic retinopathy as possible. Additionally, our definition of Parkinson’s disease was created in collaboration with and verified by a neurologist specializing in movement disorders. We also acknowledge the limitations of this study. As general practitioners do not report to The Danish National Patient Register, data from the primary sector are not included in the registry. Patients with a less severe degree of Parkinson’s disease who have not visited the hospital are therefore not labelled with an ICD-10 code and thereby not included in outcome. Concurrently, patients with a more severe degree of Parkinson’s disease may restrain from attending the eye-screening programme because of a greater disease burden. Nevertheless, this issue should be eliminated by the design of the prospective part of the study. Another limitation was the unavailable data on current smoking status and HbA1c. By creating a variable to identify potential smokers based on data on smoking characteristics from the registries, we adjusted for the possible confounding effect of smoking, but it is important to keep in mind that the variable is made as a proxy and cannot be used as a measure of the actual smoking status in Denmark. Data on HbA1c could have let us evaluate the impact of poor glycaemic control on the development of Parkinson’s disease. Additionally, earlier studies have found that statins may have a protective effect on the development of Parkinson’s disease and this could influence the results.^44^^,^^45^

In conclusion, this national register-based cohort study, including nearly half of the Danish population above age 50, found that while patients with diabetes were less likely to develop Parkinson’s disease, diabetic retinopathy did not affect the risk of prevalent or incident Parkinson’s disease. This study, therefore, does not support the hypothesis of diabetic retinopathy being an independent risk factor for Parkinson’s disease.

## Supplementary material

Supplementary material is available at *Brain Communications* online.

## Funding

This work was supported by the VELUX FOUNDATION (grant number 00028744).

## Competing interests

The authors report no competing interests.

## Supplementary Material

fcab262_Supplementary_Data

## Abbreviations

ATC =: Anatomical Therapeutic Chemical Classification System
CI =: confidence interval
DiaBase =: The Danish Registry of Diabetic Retinopathy
DNPR =: The Danish National Patient Register
HR =: hazard ratio
ICD-10 =: 10th version of the International Classification of Diseases system
OR =: odds ratio
