# Supplementary material for: Diabetic retinopathy as a potential marker of Parkinson’s disease: a register-based cohort study
Source: Brain Commun. 2021 Nov 8;3(4):fcab262. doi: 10.1093/braincomms/fcab262 (PMC8599077; doi:10.1093/braincomms/fcab262)
Supplement: fcab262_Supplementary_Data [file fcab262_supplementary_data.zip › Appendix_1.docx]

**Appendix 1**

The Danish Registry of Diabetic Retinopathy (DiaBase) is a national clinical quality database established between 2003 and 2006 with the aim of monitoring the development of diabetic eye disease in Denmark and evaluating accessibility and effectiveness of the screening program. Data on all patients above 18 years of age diagnosed with diabetes who attend the national screening program for diabetic eye disease, is reported to the registry.^21^ The registry in reports data on all patients screened at selected departments of ophthalmology and by practicing ophthalmologists. The screening program is free of charge, and all patients with diabetes are encouraged to participate. The main variables include screening unit, patient name and Civil Personal Registration number, date, indication for screening, previous eye surgery, grading, next examination date and more. The grading of diabetic retinopathy is done according to the International Clinical Diabetic Retinopathy Disease Severity Scale, which contains five stages: level 0 (no diabetic retinopathy), 1-3 (mild, moderate and severe non-proliferative diabetic retinopathy) or 4 (proliferative diabetic retinopathy).^22^

The Danish National Patient Registry (DNPR) was established in 1977 and contains information on all hospital contacts.^23^ Its primary aim is to continually monitor all hospital and health services in order to evaluate health economy, health quality assurance, hospital planning, disease and treatment frequency etc. It is compulsory for both public and private hospitals to submit data to the DNPR. The reported information includes administrative data (covering the Civil Personal Registration number, residence, hospital and department codes, admission type etc.), diagnoses (primary, secondary, supplementary, etc.), treatments and examinations. Individuals are diagnosed and coded according to the 10^th^ version of the International Classification of Diseases system (ICD-10).^24^

The National Prescription Registry was established in 1995 and contains individual-level information on prescribed medication redeemed at community pharmacies in Denmark.^20^ Information regarding the dispensed medication, such as the dispensing date and the Nordic article number identifying the unique drug package, is linked to the Civil Personal Registration number. The prescribed medication is classified in accordance with the Anatomical Therapeutic Chemical Classification System (ATC).^25^ The registry is considered to be high in validity and completeness of registration. Data is available to researchers in an anonymized form and can only be accessed through Research Service at Statistics Denmark or the Danish Health Data Authority.^20^

The Danish Civil Registration System is a registry established in 1968, containing basic personal information on gender, birth year, marital- and vital status and more.^26^ All information is linked to a unique ten-digit Civil Personal Register number, which is given to all persons who are either born of a mother already registered in the registry, have their birth or baptism registered in the Danish church register or reside legally in Denmark for three months or more. Daily updates are made on information on migration and vital status. The Danish Civil Registration System is close to complete and data accuracy is ensured by several levels of control, including law requirements of registration.
