# Supplementary material for: Diabetic retinopathy as a potential marker of Parkinson’s disease: a register-based cohort study
Source: Brain Commun. 2021 Nov 8;3(4):fcab262. doi: 10.1093/braincomms/fcab262 (PMC8599077; doi:10.1093/braincomms/fcab262)
Supplement: fcab262_Supplementary_Data [file fcab262_supplementary_data.zip › Supplementary material.docx]

**Supplementary material**

Supplementary Table 1: Determination of type of diabetes

Supplementary Table 2: Smoking variable definition list

**Supplementary Table 1**

Determination of diabetes according to International Classification of Diseases (ICD) version 10 codes for diabetes and Anatomical Therapeutic Chemical Classification (ATC) codes for treatment of diabetes.

| ICD version 10 code for diabetes* | | ATC code for diabetes treatment^†^ | | Type of diabetes |
| --- | --- | --- | --- | --- |
| DE10* | DE11* | A10A* | A10B* |  |
| Yes | No | No | No | Unknown |
| Yes | Yes | No | No | Unknown |
| Yes | No | Yes | No | Type 1 diabetes |
| Yes | No | No | Yes | Unknown |
| Yes | No | Yes | Yes | Type 1 diabetes |
| Yes | Yes | No | Yes | Unknown |
| Yes | Yes | Yes | No | Unknown |
| Yes | Yes | Yes | Yes | Unknown |
| No | Yes | No | No | Type 2 diabetes |
| No | No | Yes | No | Unknown |
| No | No | No | Yes | Type 2 diabetes |
| No | Yes | Yes | No | Type 2 diabetes |
| No | Yes | No | Yes | Type 2 diabetes |
| No | No | Yes | Yes | Type 2 diabetes |
| No | Yes | Yes | Yes | Type 2 diabetes |
| No | No | No | No | Type 2 diabetes |

The table is modified to identify patients with diabetes in the control population.

*ICD codes: DE10*: type 1 diabetes, DE11* type 2 diabetes. ^†^ATC codes for redeemed prescriptions: A10A*: insulin, A10B*: blood glucose lowering drugs excl. insulin

**Supplementary table 2**

Smoking variable definition list based on ICD-10 codes and ATC codes.

| **Diagnosis** | **ICD-10 code** |
| --- | --- |
| COPD | J44, J440, J441, J449 |
| Diseases caused by the use of tobacco | F17* |
| Smoker | ZZP01A1A |
| Daily smoking | Z720E |
|  |  |
| **Prescription** | **ATC-code** |
| Specific COPD medication | R03BB05, R03BB06, R03BB07 |
| Vareniclin | N07BA03 |
| Bupropion | N06AX12 |
|  |  |
| **Treatment** | **ICD-10 code** |
| COPD rehabilitation status | ZZP0040A*, AZAA0A |
| Smoking cessation counseling | BVDT0* |
| Treatment with pharmacology against nicotine abuse | BRHT* |
| Counselling because of tobacco abuse | Z716 |
| Urged for smoking cessation | ZZP0020 |

*Include all subsections of the respective code.

Abbreviations: ATC = Anatomical Therapeutic Chemical Classification; COPD = chronic obstructive pulmonary disease; ICD = International Classification of Diseases
